# Bacteria that Travel: The Quality of Aircraft Water

**DOI:** 10.3390/ijerph121113938

**Published:** 2015-10-30

**Authors:** Harald Handschuh, Jean O’ Dwyer, Catherine C. Adley

**Affiliations:** 1 Microbiology Laboratory, Department of Chemical and Environmental Sciences, University of Limerick, V94 T9PX Limerick, Ireland; E-Mail: harald.handschuh@ul.ie; 2 Department of Life Sciences, University of Limerick, V94 T9PX Limerick, Ireland; E-Mail: Jean.ODwyer@ul.ie

**Keywords:** aircraft, potable water, microbial contamination

## Abstract

The travelling population is increasing globally year on year. International tourist arrival figures reached 1087 million in 2013 and 1133 million in 2014; of which 53% and 54% respectively accounted for air transport. The water on board aircraft is sourced from surface or ground water; piped to a central filling point and distributed to each aircraft by water service vehicles at the home base or at the destination airport. The purpose of this study was to ascertain the microbial, chemical (pH; Total and Free chlorine) and physical (temperature) quality of water from two aircraft, long- and short-haul, as well as from the original water source and the water service vehicle. A total of 154 water samples were collected and analysed. Long-haul flights were found to be significantly poorer in terms of microbial quality than short haul flights (*p* = 0.015). Furthermore, correlation and regression analysis showed that the water service vehicle was a significant source of increased microbial load in aircraft. Microbial diversity was also demonstrated, with 37 bacterial species identified belonging to eight classes: γ-Proteobacteria; β-Proteobacteria; α-Proteobacteria; Bacilli; Actinobacteria; Flavobacteria; Sphingobacteria and Cytophaga; using phenotypic and 16*S* rDNA sequence-based analysis. We present a novel quantified study of aircraft-related potable water supplies.

## 1. Introduction

International tourist numbers worldwide have grown significantly since the middle of the 20th century, with 1087 million travelers in 2013 alone, 53% of which travelled via air transport, as seen in Table 1. In the same year, the commercial air transport industry comprised 1397 airlines with 36.4 million scheduled flights from 3864 airports [1]. This trend is set to increase further, with forecasted growth of 3%–4% for 2015 and up to 1,800 million arrivals by 2030 (Table 1) [2].

**Table 1:** International Tourism Arrivals Statistics 1950 to 2030.

International Tourism Arrivals			
Year	Overall (Millions)	Air Travel	Reference
1950	25	NA	[2]
2000	674	NA	[2]
2010	949	51%	[2]
2013	1087	53%	[3]
2014	1133	54%	[2]
2015	3%–4% Growth Forecast		[2]
2030	1800 Forecast		[2]

Abbreviation: NA, data not available.

As a result of the number and volume of airline passengers, factors affecting public health are an area of concern with regard to in-flight safety. For the purpose of this paper, the focus is placed on the quality of the on-board water supply. The potable water stored on an aircraft is consumed by passengers in multiple ways, for example, as a component of hot beverages and soups, for reconstituting baby food, or swallowed simply as a glass of water for the intake of medicine. Indirect contact with water is also prevalent via hot towels for refreshing purposes, in rinsing food equipment, for washing hands, teeth and of course for flushing toilets. Therefore, the quality of water on board an aircraft is an area of importance in terms of public health.

Water, by its very nature, can host and support an array of microorganisms, both pathogenic [4], and non-pathogenic, including bacteria, fungi, viruses, protozoa and schistosoma [5]. Bacteria found in water include natural aquatic, soil-resident and human or animal intestinal bacteria. Natural aquatic bacteria are chiefly Gram-negative and include *Pseudomonas*, *Flavobacterium*, *Cytophaga*, *Acinetobacter* and *Chromobacterium*. Gram-positive bacteria include *Micrococcus*, *Bacillus* and coryneform bacteria [6,7].

With regard to aircraft potable water, the tanks on board are the responsibility of the airline carrier, and are typically topped up, if required, at any airport, although domestic or European-bound aircraft generally refill at the home airport to avoid costs. Therefore, water consumption is estimated for each round trip by the airline’s fuel saving committee. Water tanks are rarely emptied completely and are generally only emptied and refilled if the water system is serviced, the water on board has been entirely consumed, or during cold winter days while the aircraft is not in operation, to avoid the system freezing. Like all drinking water supplies, aircraft potable water is regulated under European and international legislation. At EU level, *European Council Directive* 98/83/EC legislates total viable count (TVC) limits at 100 cfu/mL at 22 °C and 20 cfu/mL at 37 °C, and other microbiological limits for drinking water in containers at 0 cfu/250 mL for *E. coli*, *Enterococci* and *Pseudomonas aeruginosa* [8], and *Regulation (EC)* No. 178/2002 demands regular monitoring of set parameters to evaluate the potable quality of water, which include *Escherichia coli* and *Enterococci* at zero colony forming units (cfu)/100 mL [9]. In the United States of America (US), drinking water is legislated under the Safe Drinking Water Act (SDWA), which was established by the United States Environmental Protection Agency (US EPA) in 1974, and revised in 1986 and 1996. Furthermore, the US EPA demands that water system operators must be certified to ensure competency, and issued guidelines in 1999 outlining the minimum standards for this type of certification [10]. The US EPA also developed the National Primary Drinking Water Regulations (NPDWR) under the SDWA, in which mandatory water quality standards are outlined that apply to the public water systems. The NPDWR contain parameters for monitoring of disinfectants, disinfectant by-products, inorganic and organic chemicals, radionuclides and microorganisms as regards Maximum Contaminant Level (MCL), which is the maximum allowable limit and Maximum Contaminant Level Goal (MCLG) defined as a non-enforceable limit [11].

For this study, the particular area of interest is the microbiological quality of aircraft water, as pathogenic organisms are both highly infectious and commonly contagious. In review of relevant literature, a 2002 survey conducted by reporters from The Wall Street Journal, on the microbial content of airline water [12] documented some “alarming” results: samples taken from galleys and lavatory taps on 14 different flights by 10 different airlines were found to contain *Pseudomonas aeruginosa*, *Citrobacter*, *Acinetobacter junii-genospecies 5*, *Sphingomonas echinoides*, *Salmonella*, *Staphylococcus* and eggs of aquatic insects. However, it must be noted that their experimental approach was criticised by the airline industry, as the methods employed were evaluated as questionable [13]. During 2004, the US EPA collected water samples from 327 national and international commercial aircraft on which microbiological analysis was carried out. Some of the results did not meet US EPA’s NPDWR standards, with 21% of the aircraft containing no residual chlorine and total coliforms found in 15% of samples, of which 4.1% tested positive for *E. coli* [14]. In 2006, Health Canada conducted a similar study, collecting 431 water samples and found that 15.1% of the aircraft had tested positive for Total Coliforms and 1.2% for *E. coli*. At the same time, this government department informed air travellers with low immune systems to avoid hot and cold beverages in which tap water was a component [15]. In light of these findings, the Air Transport Association (ATA) responded with a statement to underline its confidence in airline drinking water and its commitment to working with the US EPA. Nevertheless, the ATA expressed its uneasiness at the sample results, which were representative of approximately 1% of the worldwide fleet. The results appeared to contradict a study carried out by the Food and Drug Administration (FDA) and ATA’s own study which clearly showed that none of their samples tested positive for *E. coli*. The ATA raised concerns with regard to EPA sampling procedures in particular, as the latter had included samples from on-board toilet facilities, where a high risk exists for cross-contamination of the samples taken. The International Air Transport Association (IATA) also issued a recommendation to airlines, advising that they put in place independent monitors to ensure any EPA sampling procedures met approved standards [16]. In 2005, the US EPA in consultation with the ATA developed the Aircraft Drinking Water Rule (ADWR), implemented in 2011, specifically for the airline industry to ensure the provision of safe drinking water for passengers and flight-crew, as it was not deemed feasible to apply the NPDWR to aircraft water systems, which are mobile and dissimilar in design compared with land-based water distribution systems. The ADWR requires that microbiological tests only be carried out for Total Coliforms and *E. coli*, which are required to be absent and if detected public access to aircraft water must be restricted within 24 h of receiving positive results from the laboratory. The ADWR also covers best management practice, corrective actions, public notification, operator training, reporting and recording. The frequency of sanitising and flushing of the aircraft water system is linked with water sampling and analysis for coliforms and *E. coli* [17]. The “acute” total coliform MCL violation under the 1989 Total Coliform Rule (TCR) has been maintained as the MCL for *E. coli* under the Revised Total Coliform Rule (RTCR). Public water systems (PWS) and primacy agencies must comply with the requirements of the RTCR by 1 April 2016. Until then, PWS and primacy agencies must continue to comply with the 1989 TCR [18].

The purpose of this study was to assess the quality of airline water through sampling of both long- and short-haul aircraft, the on-board galleys, the water service vehicle and the original water source. Through the use of statistical analysis, assessment of variance across sampling locations was derived and correlations between chemical and physical water parameters and microbiological quality analysed. The aim of this study was to provide a quantitative overview of on-board potable water quality and possible areas that require intervention to safeguard public health.

## 2. Experimental Section

### 2.1. Sampling Locations and Frequency

Two water sampling activities (A and B) were conducted over a 133-day period between September 2010 and February 2011, during which a total of 154 samples were collected. The sampling periods are represented in Table 2 and Table 3. Sampling was carried out using standard procedures for water sampling: BS ISO 5667-21:2010 [19] and ISO 5667-5:2006 [20]. Prior to flushing and sampling, the taps and hoses were disinfected using Clinical Alcohol Wipes (Pal International, Leicestershire, UK). All galley taps on the aircraft have a flow rate of 3 L per minute and were flushed for 3 min. The water service vehicle has a flow rate of 15 L per minute and the Water Source has a flow rate of approximately 120 L per minute and both were flushed for 30 s. For both sampling activities (A and B), two 250 mL samples were collected in pre-sterilised wide-mouthed sample containers (Sterilin^®^, Gwent, UK) from each of the sample points.

In sampling activity A, water samples were taken from two particular aircraft: one designated for long-haul flights with a dual water tank capacity of 700 L (350 L per tank) and one from the short-haul allocated fleet (Table 2) with a single water tank capacity of 300 L. A total of 90 samples were collected over a 15-week time frame from both the forward and aft galleys. In sampling activity B, the same long-haul aircraft was selected and the aircraft water was sampled on a daily basis, collecting 64 samples in total (Table 3). Water samples were also collected from the airport water source, which was the water collection point for all aircraft water service vehicles at the time. Water service vehicles are mobile water tankers, each with a potential capacity of 3000 to 5000 L of water, which transport water from the water source to aircraft. The water samples were collected directly from the water service vehicle fill hose which is connected to the aircraft during refilling of the potable water tank on board. All samples were transported at temperatures between 2 °C and 8 °C from the sampling site to the laboratory and analysed within one hour. During sampling activity A and B the long-haul aircraft remained on the same flight schedule for long-haul destinations and water was uplifted from the same European airport and North American east coast airports. Likewise, the short-haul aircraft remained on the multi-sector central European flight schedule during sampling activity A.

**Table 2:** Water Sampling Activity A/Location, Number of Samples and Sampling Frequency.

Sampling Activity A		
Sampling Period 2 September 2010–16 December 2010 (105 Days/15 Weeks)		
Location	Number of Samples	Sampling Frequency
Long-haul AC, Fwd & Aft	30	Weekly
Short-haul AC, Fwd & Aft	30	Weekly
Water Service Vehicle	15	Weekly
Water Source	15	Weekly

Abbreviations: Fwd, forward galley; Aft, aft galley; AC, aircraft.

**Table 3:** Water Sampling Activity B/Location, Number of Samples and Sampling Frequency.

Sampling Activity B		
Sampling Period 10 January 2011–7 February 2011 (28 Days/4 Weeks)		
Location	Number of Samples	Sampling Frequency
Long-haul AC, Fwd & Aft	56	Daily
Water Service Vehicle	4	Weekly
Water Source	4	Weekly

Abbreviations: Fwd, forward galley; Aft, aft galley; AC, aircraft.

### 2.2. Water Quality Analysis

Sampling was undertaken when the aircraft returned from their respective destinations. The Fuel Saving Committee stipulated that water levels should be set at 100% in the case of long-haul aircraft and at 75% for short-haul aircraft prior to departure of any aircraft. The water level of the aircraft tanks was recorded prior to water sampling by observing the water level indicator on the flight attendant panel (FAP) in the Fwd galley on board aircraft. Sampling was not always possible, for logistical reasons, directly upon aircraft arrival, as refilling had sometimes already taken place. Water level readings were therefore essential in order to determine whether bacterial populations were related to airport water supplies.

The water temperature was measured from each sample source prior to taking any samples by placing a calibrated core temperature probe PT100 platinum sensor of a digital thermometer (Delta OHM, Caselle di Selvazzano PD, Italy) under the running water stream. The readout on the instrument was recorded once it became constant. All temperature results were recorded as degrees Celsius (°C). The pH of the collected water samples were measured, applying standard procedure BS EN ISO 10523:2010 [21] and using pH meter (Hanna pH210, Hanna Instruments, Laval, QC, Canada) and probe (Hanna HI 7669/2W), enabling the automatic temperature compensation feature on the instrument. Total and free chlorine were determined by using a photometer (Tintometer GmbH, Lovibond, Dortmund, Germany) and applying standard procedure BS EN ISO 7393-2:2000 [22]. The results were recorded as mg/L free chlorine (fCl) and mg/L total chlorine (tCl). Acceptable pH limits of between 6.5 and 9.5 and residual chlorine levels of between 0.3 mL/L and 0.8 mL/L in aircraft drinking water are recommended by the International Air Transport Association (IATA) Drinking-Water Quality Pool (IDQP) [23].

### 2.3. Microbiological Analysis

Total viable counts (TVC) were based on ISO 6222:1999 [24]. Serial dilutions [25] were performed and Yeast Extract Agar (YEA) (Oxoid) was inoculated by the pour plate technique in duplicate with 1 mL undiluted, 10^−2^, 10^−3^ of water sample. The inoculated plates were incubated at 37 °C for 44 ± 4 h and the duplicate petri dishes at 22 °C for 68 ± 4 h. The colonies were counted with the use of a digital colony counter (J.P. Selecta, S.A. Barcelona, Spain). The results were expressed as colony forming units per millilitre (cfu/mL). For quality control purposes 1 mL of Tryptone SEL Bouillon diluent (AES Laboratoire, Bruz Cedex, France) was added in duplication to 15 mL of YEA in a Petri dish and incubated at 37 °C and 22 °C, with each batch of samples tested. Bacterial colonies were randomly picked from the petri dishes of aircraft, water service vehicle and source water and streaked onto a YEA plate, inverted and incubated for three to five days at the same temperature as the original plate from which the colony originated. A total of 196 isolates were further purified to single colonies and were identified by Gram staining, oxidase and catalase testing [26] and were subsequently subjected to the automated biochemical test VITEK (BioMérieux) or the manual biochemical test Analytical Profile Index (API) 20NE (BioMérieux). Organism matches were expressed in percentages by the VITEK and API databases. Whenever results were inconclusive with either phenotypic method, the 16*S* ribosomal RNA (rDNA)-based identification system MicroSeq500^®^ (Applied Biosystems™, Waltham, MA, USA) was employed by the contract laboratory (Eurofins, Dungarvan, Ireland).

The water samples obtained were analysed for Coliforms and *E. coli* using Colilert-18 and Enterolert-E (IDEXX) for the detection and quantification of *Enterococci* following manufacturer’s instructions, which are US EPA approved methods [27]. Results were determined by reference to the IDEXX Most Probable Number (MPN) Table and recorded as MPN per 100 mL. For quality control purposes, to test the reliability of the reagent, a negative control containing sterile water was processed with the IDEXX system with every batch of samples with expected results of no colour change in any of the wells.

### 2.4. Statistical Analysis

Prior to statistical analysis, predictor variables were assessed for normality using the Shapiro-Wilk test; the distribution of the data was utilised for selection of the appropriate statistical analysis method. All data displayed a non-normal distribution (*p* ≤ 0.05) and thus non-parametric analyses were applied. Univariate analyses were undertaken using One-Way ANOVA and Mann-Whitney U analysis or using Spearman’s Correlation Coefficient, as appropriate. Linear regression (LR) models were constructed using the total viable counts in the water sample as the dependent variable. For consistency and clarity, the dependent variable was categorised under three different scenarios over two incubation periods of 22 °C and 37 °C. The “enter” method with backward elimination of predictor variables was used for Linear Regression (LR) model development. The adjusted R^2^ (Goodness of Fit) was used to estimate effect size. SPSS^®^ version 22 (IBM Corporation, New York, NY, USA) was employed for all statistical analyses and the significance level was set at 95% (*p* ≤ 0.05) for all analyses.

## 3. Results and Discussion

### 3.1. Physical and Chemical Analysis

Prior to analysis, aircraft water tank levels were measured. During sampling activity A, the water tank levels for the long-haul aircraft (*n* = 30) ranged from 50% to 100%, achieving a mean value of $\bar{x}$ = 79%, Standard Error (S.E) = 1.73, and for the short-haul aircraft (*n* = 30) the level varied between 35% and 100% ($\bar{x}$ = 72.1% S.E = 2.42). This fluctuation in water level demonstrates that a mixture of water samples were taken, some prior to and some post-filling at the home-base for long-haul aircraft and predominantly post-filling for short-haul aircraft. Physical and chemical analyses were performed on source water, the water service vehicle and both the long- and short-haul aircraft carriers for sampling activity A, the results of which are shown in Table 4.

**Table 4:** Physical and Chemical Analysis for Sampling Activity A across Long- and Short-haul Aircraft, the Water Source and the Water Service Vehicle.

Sampling Activity A								
	Long-Haul		Short-Haul		Water Source		Water Service Vehicle	
	Mean	S.E	Mean	S.E	Mean	S.E	Mean	S.E
pH	7.74	0.01	7.71	0.02	7.70	0.03	7.70	0.02
fCl (mg/L)	0.01	0.00	0.01	0.00	0.09	0.02	0.09	0.01
tCl (mg/L)	0.02	0.00	0.03	0.00	0.13	0.02	0.13	0.01
Temperature °C	17.41	0.34	16.12	0.64	13.24	0.64	13.24	0.37

Abbreviations: S.E, Standard Error of Mean; fCl, Free Chlorine; tCl, Total Chlorine.

Variance between the physical and chemical properties was apparent in sampling activity A, in particular with regard to temperature and chlorine levels, which were higher in the water source and the water service vehicle. To quantifiably assess the differences across groups, a one-way ANOVA was conducted to compare the effect of sampling location on the results of mean pH, Free Cl, Total Cl and Temperature, across the sampling locations. There was a significant difference across group means for three independent variables at the *p* < 0.05 level. Total Chlorine (F = 42.683, *p* = 0.001), Free Chlorine (F = 42.683, *p* = 0.001) and Temperature (F = 18.941, *p* = 0.001), demonstrated significant differences across the sampling locations. Conversely, pH (F = 1.057, *p* = 0.367) showed little variance across group categories.

For sampling activity B, samples were taken daily from the long-haul aircraft; the results are shown in Table 5. Similar to sampling activity A, variance was evident across sampling locations with reference to temperature and chlorine levels.

A one-way ANOVA was also conducted on the sampling activity B results. Interestingly, during sampling activity B, there was a significant difference across group means, for all independent variables at the *p* < 0.05 level. Total Chlorine (F = 42.683, *p* = < 0.001), Free Chlorine (F = 42.683, *p* = <0.001), Temperature (F = 18.941, *p* = < 0.001) and pH (F = 7.904, *p* = < 0.001) demonstrated significant differences across the sampling locations. This could be evidence that subtleties in pH levels may be more representative through daily rather than weekly/monthly sampling.

**Table 5:** Physical and Chemical Analysis for Sampling Activity B across the Long-Haul aircraft, the Water Source and the Water Service Vehicle.

Sampling Activity B						
	Long Haul		Water Source		Water Service Vehicle	
	Mean	S.E	Mean	S.E	Mean	S.E
pH	7.63	0.01	7.50	0.03	7.53	0.02
fCL	0.06	0.00	0.35	0.11	0.20	0.05
tCL	0.11	0.01	0.41	0.11	0.27	0.05
Temperature	13.53	0.28	8.18	0.27	8.45	0.66

Abbreviations: S.E, Standard Error of Mean; fCl, Free Chlorine; tCl, Total Chlorine.

The most striking difference across the sampling sites was with regard to temperature, with mean temperatures varying by more than 5 °C. The difference in temperature across the groups for sampling activity B is likely indicative of the temperature differential associated with this sampling activity. Sampling activity B was conducted in January, a winter month in the sampling location. As a result, it was expected that all outside water sources would have a significantly lower temperature than those on board an aircraft. In both sampling activities, the chlorine levels in the water service vehicle and the water source were significantly higher than in the on-board water tanks themselves, with chlorine levels from the water source in sampling activity B in breach of IDQP guidelines (>0.3 mg/L). This was reported to the airport authority and it was later identified as being due to a faulty chlorine dosing system at the airport reservoir. The chlorine levels did decrease, while travelling from the water service vehicle ($\bar{x}$ = 0.27) to the aircraft ($\bar{x}$ = 0.11), indicating chlorine dissociation along the supply chain of drinking water from the water source to the on-board taps.

Analysis was also conducted to measure any differences between the galleys on board both long- and short-haul aircraft (Table 6). On the short-haul aircraft water is supplied to both rear (Aft) and front (Fwd) galleys from one water tank located in the middle of the aircraft, but through separate water lines. On the long-haul aircraft there are two water tanks located towards the tail of the plane but with separate water pipes; water is drawn from both tanks at the same time. Significant differences across these sampling locations could be an indication of a problem within the water distribution system.

**Table 6:** Physical and Chemical Analysis for the Galleys across the Long-Haul Aircraft, the Water Source and the Water Service Vehicle.

	Sampling across Galleys							
	Long-Haul				Short-Haul			
	Fwd		Aft		Fwd		Aft	
	Mean	S.E Mean	Mean	S.E Mean	Mean	S.E Mean	Mean	S.E Mean
pH	7.67	0.01	7.66	0.01	7.66	0.03	7.76	0.03
fCL	0.05	0	0.04	0.01	0.01	0	0.01	0
tCL	0.1	0.01	0.06	0.01	0.04	0.01	0.02	0.01
Temp	15.44	0.41	14.33	0.31	16.11	0.85	16.13	0.98

Abbreviations: S.E, Standard Error of Mean; fCl, Free Chlorine; tCl, Total Chlorine, Temp, Temperature.

Upon observation of the descriptive statistics for both aircraft, subtle variances in both temperature and chlorine (free and total) can be observed across the galleys (Fwd and Aft). For example, for long-haul flights the mean total chlorine between galleys varied by 0.04 mg/L.

In order to quantifiably assess differences between the galleys across the two aircraft, a Mann Whitney U test for differences of means was employed, using Galley as the dependent variable and the chemical and physical analysis results (pH, fCl, tCl, temperature) as the independent variables. For the short-haul aircraft, there was no significant difference between the Fwd and the Aft galleys as shown in Table 7. For the long-haul aircraft (Table 7), significant difference in means was found between the galleys for two predictor variables: Total Chlorine (U = −2.256, *p* = 0.024) and Temperature (U = −0.987, *p* = 0.047). These results suggest that where more than one water tank is utilised, the chemical and physical composition of the water is more likely to differ between galleys and this could be an important consideration in water distribution system design.

**Table 7:** Mann Whitney U test for differences of means across physical and chemical analysis results for long- and short-haul aircraft, using Galley (Fwd or Aft) as the independent variables.

	Variable Name *	Test Statistic (U)	Significance (*p*)
**Short-Haul**	pH	1.474	0.140
	Free Chlorine	−0.507	0.713
	Total Chlorine	−1.239	0.215
	Temperature	0.062	0.969
**Long-Haul**	pH	1.474	0.782
	Free Chlorine	−1.852	0.064
	Total Chlorine	−2.256	0.024
	Temperature	−0.987	0.047

* , Independent Variable is Galley (Fwd or Aft); U, Mann Whitney U analysis.

### 3.2. Microbial Quality of Airline Water

Assessment of the microbial quality of airline water was carried out via the enumeration of Total Viable Counts (TVC). The results of sampling activities A and B, detailing the long- and short-haul (sampling activity A only) aircraft, the water service vehicle and the water source are detailed in Table 8. Generally, it is noted that the bacterial quality of the water diminishes greatly between the water source and the water service vehicle, increasing from $\bar{x}$ = 700 cfu/mL at source for long-haul flights at 22 °C for sampling activity A, to $\bar{x}$ = 140,000 cfu/mL in the water service vehicle; a 200-fold increase. This highlights the importance of routine inspection and cleaning of intermediate water holding vessels such as the water service vehicle.

Of particular interest in this study was the assessment of any differences in microbiological quality across aircraft, *i.e.*, long- and short-haul aircraft and also within galleys on each aircraft. To assess this, a Mann Whitney U analysis was performed. It was discovered that there is a significant difference (χ^2^ = 2.432, *p* = 0.015) between the means of TVCs in long-haul (Mean rank = 35.98) and short-haul (Mean rank = 25.02) aircraft, for samples incubated at 37 °C in sampling activity A. For samples incubated at 22 °C, there was not a significant association (χ^2^ = 1.730, *p* =0.084) at the 95% confidence level, however as it is approaching significance, this should be revaluated with more data. This is an important result, as it suggests that long-haul aircraft require more stringent upkeep in terms of water quality in order to safeguard public health.

**Table 8:** Microbiological analysis for sampling activity A and B across the aircraft, the source water and the water service vehicle.

				TVC			
				Mean	Max	Min	S.E
**Sampling Activity**	**A**	Long-Haul AC	37 °C	12,010.33	59,000.00	0.00	2337.35
			22 °C	7973.40	32,200.00	0.00	1311.01
		Short-Haul AC	37 °C	4835.17	15,600.00	1.00	828.55
			22 °C	5173.70	20,900.00	5.00	953.30
		Water Source	37 °C	0.27	2.00	0.00	0.15
			22 °C	85.33	700.00	0.00	56.22
		Water Service Vehicle	37 °C	229.47	1300.00	0.00	105.20
			22 °C	11,642.87	140,000.00	0.00	9211.88
	**B**	Long-Haul AC	37 °C	17,715.18	150,000.00	450.00	3093.91
			22 °C	15,988.93	96,800.00	450.00	2153.76
		Water Source	37 °C	0.00	0.00	0.00	0.00
			22 °C	4.00	16.00	0.00	4.00
		Water Service Vehicle	37 °C	0.00	0.00	0.00	0.00
			22 °C	7.25	29.00	0.00	7.25

Abbreviations: AC, aircraft.

In terms of TVC counts across the galleys, unlike chemical properties, there was no significant difference found between the Fwd and Aft galleys on any aircraft, at any incubation temperature across both sampling activities, and this could be due to the rapid growth and distribution of microorganisms within the system.

**Figure 1:**
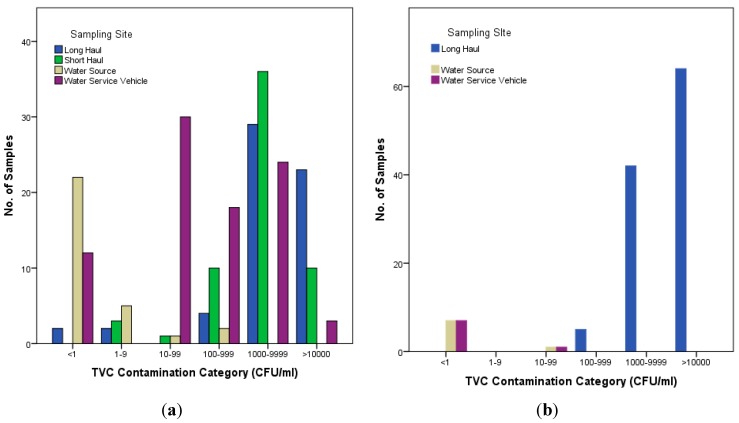
The distribution of TVC contamination categories by Sampling Activity. (**a**) Sampling Activity A; (**b**) Sampling Activity B.

Further to the assessment of mean total viable count (TVC) values, TVCs were also categorised based on severity into six categories (CFU/mL) as follows: <1, 1–9, 10–99, 100–999, 1000–9999 and >10,000 CFU/mL. The purpose of this categorisation was to highlight sampling sites which had consistently higher TVC counts, information that can be lost when focusing only on mean values due to outliers. The samples were then segregated into two incubation temperature groups: samples incubated at 22 °C and 37 °C for both sampling activities as shown in Table 9 and Figure 1. For the water source, in both sampling activities A and B the majority of samples fall into the <1 cfu/mL (*n* = 29) and the 1–9 cfu/mL category (*n* = 5) demonstrating that the source water, which is from a treated water supply, is of high quality. However, two results obtained during sampling activity A were categorised as 100–999 cfu/100 mL, and thus did not conform to *European Council Directive 98/83/EC* set limits. Those particular TVC results coincided with low fCl (0.0 mL/L–0.05 mL/L) recordings for the same samples, highlighting the effect of low fCl levels on bacterial growth in water. For the water service vehicle, particularly in sampling activity A, there is a more varied distribution across the contamination categories with 10 samples falling into 10–99 cfu/mL, 6 into 100–999 cfu/mL and 8 into the 1000–9999 cfu/mL category across both incubation temperatures. This again highlights the importance of routine inspection and cleaning of intermediate water holding vessels.

**Table 9:** The distribution of TVC results by contamination category.

				TVC Category					
				<1	1–9	10–99	100–999	1000–9999	>10,000
**Sampling Activity**	**A**	Long-Haul	37 °C	1	1	0	2	13	13
			22 °C	1	1	0	2	16	10
		Short-Haul	37 °C	0	2	0	5	18	5
			22 °C	0	1	1	5	18	5
		Water Source	37 °C	12	3	0	0	0	0
			22 °C	10	2	1	2	0	0
		Water Service Vehicle	37 °C	2	0	8	3	2	0
			22 °C	2	0	2	3	6	1
	**B**	Long-Haul	37 °C	0	0	0	3	21	31
			22 °C	0	0	0	2	21	33
		Water Source	37 °C	4	0	0	0	0	0
			22 °C	3	0	1	0	0	0
		Water Service Vehicle	37 °C	4	0	0	0	0	0
			22 °C	3	0	1	0	0	0

For the aircraft, both long- and short-haul across both sampling activities, we see a shift in category distribution towards the higher TVCs. For sampling activity A, 29 and 36 samples fall into the 1000–9999 cfu/mL category for long-haul and short-haul aircraft respectively. In sampling activity B, of the samples tested 64 were in the >10,000 cfu/mL range. These results demonstrate that aircraft water supply tanks are conducive for microbial growth.

In addition to TVC analysis, the water samples were also analysed for the presence of Coliforms, *E. coli* and *Enterococci*, which are indicators of serious microbiological contamination. There was no presence of any species detected with the IDEXX system in any of the water samples taken during sampling activities A and B; this absence demonstrated compliance with regulatory standards. This is a particularly important result in terms of acceptable quality of the water.

### 3.3. Microbiological, Physical and Chemical Correlation Coefficients and Regression Analysis

Subsequent to the chemical, physical and microbial analysis results, the relationship between these characteristics and the growth of microorganisms was investigated. In particular, and as highlighted previously, an investigation into the relationship between the water service vehicle and on-board TVC counts was investigated, as well as the relationship between on-board physical and chemical characteristics and TVC.

For this evaluation, correlation statistics were derived using Spearman’s Correlation Coefficient. Samples were segregated based on incubation temperature and not haul (long- and short-), as both aircraft are filled via the same water service vehicle. The dependent variable of interest was TVC on both long- and short-haul flights and the independent variables were: Temperature, pH, Free Chlorine, Total Chorine for the on-board water samples and the same variables for the water service vehicle with the addition of TVC of same.

For sampling activity A, significant, moderate correlations were found to exist between aircraft TVC at 37 °C and the independent variables: Temperature (*r* = 0.321, *p* = 0.012), the Temperature of the water service vehicle water (*r* = 0.330, *p* = 0.010) and the TVC of the water service vehicle (*r* = 0.325, 0.011). Similarly, for TVC at 22 °C, the same three independent variables were also shown to be significantly correlated as follows: Temperature (*r* = 0.317, *p* = 0.014), Temperature of the water service vehicle water (*r* = 0.280, *p* = 0.031) and the TVC on the water service vehicle (*r* = 0.328, 0.011).

For sampling activity B, there was no correlation between water service vehicle TVC and aircraft TVC, but a significant correlation was discovered between both aircraft water temperature (*r* = 0.274, *p* = 0.003) and the water service vehicle temperature (*r* = 0.207, *p* = 0.029), as with sampling activity A. Interestingly, a significant negative correlation was found to exist between aircraft TVC and chlorine levels, both free (*r* = −0.171, *p*= 0.042) and total (*r* = −0.171, *p* = 0.042). This may be an explanatory factor with regard to the loss of significance of water service vehicle TVC. As mentioned previously, a chlorine dosage malfunction occurred at the water source for sampling activity B with a higher concentration of chlorine added. The raised chlorine levels would undoubtedly have an effect on water service vehicle TVC levels.

Further to correlation analysis, Linear Regression models were developed to establish if aircraft TVC can be predicted by the significant correlation variables namely: Temperature and water service vehicle temperature and TVC. In total two models were created for sampling activity A and using backward elimination, one variable (water service vehicle TVC) was utilised as the predictor variable in both models as shown in Table 10.

**Table 10:** Linear Regression coefficients for two Linear Regression models for sampling activity A.

Predictor Variable	B	R^2^	*p*
Water Service Vehicle (TVC) ^a^	1.055	0.297	0.021
Water Service Vehicle (TVC) ^b^	7.805	0.304	0.018

Abbreviations: ^a^ , 37 °C; ^b^ , 22 °C; B, Standard coefficient; R^2^ , Goodness Fit; *p*, significance.

At an incubation temperature of 37 °C, the simple regression showed that for every increase in TVC (cfu/mL) in the water service vehicle, an increase of 1.055 was predicted (ANOVA F =3.355, *p* = 0.021) for the aircraft TVC (cfu/mL). For incubation at 22 °C, it was predicted (ANOVA F= 5.910, *p* = 0.018) that for every one unit increase in TVC (cfu/mL) in the water service vehicle, an increase of 7.805 was predicted for the aircraft TVC (cfu/mL). These results signify that the microbiological integrity of the intermediate water vessel is paramount to the integrity of the on-board microbiological quality.

### 3.4. Isolated and Identified Bacteria

A total of 37 positively identified species were isolated during sampling activity A and B from water samples taken from long-haul and short-haul Fwd and Aft aircraft galleys, the water service vehicle tank and water source which can be seen in Table 11. The identified bacteria belong to eight classes, two of which, Bacilli and Actinobacteria, are Gram-positive and the remainder of which, γ-Proteobacteria; β-Proteobacteria; α-Proteobacteria, Flavobacteria; Sphingobacteria and Cytophaga, are Gram-negative. The 16*S* rDNA genes sequences for 22 isolates signified by “*” were identified through the MicroSeq500^®^ system, with the remaining 15 isolates identified through biochemical analysis, indicated with “^” in Table 11.

Several species among the isolated γ-Proteobacteria have been reported in literature in nosocomial settings, such as *Pseudomonas aeruginosa* [28], blood stream infections from *Pseudomonas fluorescens* [29], and *Stenotrophomonas maltophilia* associated with respiratory infections [30]. The β-Proteobacteria isolates also include reported opportunistic pathogens, for example *Alcaligenes faecalis* associated with respiratory infections in chickens [31] and postoperative infections in patients [32]. They also included nosocomial isolations of *Ralstonia paucula* formerly known as CDC group IVC-2 [33], *Burkholderia pseudomallei* described as a causative agent of melioidosis [34], *Comamonas acidovorans* causing endocarditis [35] and urinary tract infection [36]. *Neisseria flavescens* has been associated with necrotizing pneumonia and empyema [37] and *Ralstonia pickettii* reported as a causative agent in nosocomial infections [38]. α-Proteobacteria includes *Brevundimonas vesicularis* which has been reported to have caused liver abscesses in immunocompetent patients [39] and *Ochrobactrum anthropic* associated with pyogenic infections [40]. Bacteraemia has been caused in clinical settings by *Roseomonas* genomospecies [41] and *Sphingomonas paucimobilis* [42]. Within the Bacilli isolates *Bacillus thuringiensis* [43] and *Streptococcus mitis* [44] have been previously implicated in causing bacteraemia in immunocompromised patients. Within the Actinobacteria class, reported health issues of isolated bacteria include endophthalmitis associated with *Microbacterium aurum* [45], intestinal pulmonary inflammation involving *Microbacterium liquefaciens* in hospital patients after heart transplants [46]. *Rhodococcus fascians* is reported to be a phytopathogen causing leafy gall disease [47]. The Flavobacteria isolations included *Chryseobacterium indologenes* that have reportedly caused nosocomial infections in immunocompetent patients [48]. Species within the Sphingobacteria class causing clinical infections such as *Sphingobacterium multivorum* has been reported to cause septicaemia [49].

**Table 11:** Bacteria isolated from aircraft sampling activity A and B, across long-haul, short-haul, and water service vehicle and water source.

α-Proteobacteria	β-Proteobacteria	γ-Proteobacteria	Other
*Brevundimonas vesicularis* ^	*Alcaligenes faecalis* ^	*Acinetobacter haemolyticus* ^	Bacillus thuringiensis * (Bacilli)
*Caulobacter henricii* *	*Acidovorax temperans* *	*Pseudomonas aeruginosa* *	*Streptococcus mitis ^ (*Bacilli)
*Ochrobactrum anthropi* ^	*Burkholderia pseudomallei* ^	*Pseudomonas asplenii* *	*Microbacterium aurum* * (Actinobacteria)
*Roseomonas genomospecies* *4* *	*Comamonas acidovorans* *	*Pseudomonas boreopolis* *	*Microbacterium liquefaciens* * (Actinobacteria)
*Sphingobium cloacae* *	*Comamonas testosteroni* *	*Pseudomonas fluorescens* *	*Rhodococcus fascians* * (Actinobacteria)
*Sphingobium xenophagum* *	*Neisseria flavescens* *	*Pseudomonas luteola* ^	Chryseobacterium indologenes ^ (Flavobacteria)
*Sphingomonas aerolata* *	*Pelomonas saccharophila* *	*Pseudomonas stutzeri* ^	*Sphingobacterium multivorum* ^ (Sphingobacteria)
*Sphingomonas aurantiaca* *	*Ralstonia paucula* ^	*Moraxella spp* ^	*Arcicella rosea* * (Cytophagia)
*Sphingomonas paucimobilis* ^	*Ralstonia pickettii* ^	*Stenotrophomonas maltophilia* ^	
*Sphingopyxis alaskensis* *	*Variovorax paradoxus* *		

Abbreviations: * , isolates were identified through the MicroSeq500^®^ system; ^ , isolates through biochemical analysis.

## 4. Conclusions

This study analysed the quality of water from two aircraft: long- and short-haul as well the water service vehicle and the water source. Results for both sampling activities (A and B) demonstrated that the physical and chemical properties of water sampled differed between sampling locations. In particular, higher levels of chlorine (free and total) were discovered in the water service vehicle and the water source than on the aircraft itself. In sampling activity B, chlorine levels in breach of the IDQP guidelines were discovered and a follow-up investigation identified a malfunction in chlorine dosing at the water source. On board the aircraft, differences between galleys (Fwd and Aft) were found to exist for the chemical properties of the water, but the microbiological results showed no significant difference. Upon analysis of the microbiological quality of source and water service vehicle water, it was noted that TVCs increased greatly between the source and the service vehicle. This result was seen consistently over the study period and is indicative of the importance of routine inspection and cleaning of intermediate water holding vessels for example the water service vehicle. Focusing on the aircraft (long- and short-haul), a significant difference in relation to the microbiological quality was discovered, with long-haul aircraft displaying a poorer microbial quality. This is an important result as it suggests that long-haul aircraft require more stringent upkeep in terms of water quality, inclusive of the intermediate water supplier at any given location, in order to safeguard public health.

Subsequent to the general evaluation of aircraft related quality, a more in-depth statistical analysis was employed to investigate relationships between the physical and chemical properties of water (both on-board and the water service vehicle), the microbial load of water from the water service vehicle and the level of TVCs on the aircraft. Statistical correlations demonstrated that the temperature of both the aircraft water and the water service vehicle, as well as the TVC of the water service vehicle, had a significant positive relationship with on-board TVC levels. Linear regression models were then developed to assess if any of the variables could predict on-board TVC levels. The final regression models show that the TVC level of the water service vehicle can be utilised to predict the TVC on-board aircraft. For each increase of one cfu/mL of the TVC in the water service vehicle, the TVC on board the aircraft increased by a factor of 1.055 and 7.805, for 37 °C and 22 °C, respectively.

The results of this study demonstrate the variance of microbial quality across aircraft-related sampling locations and provide quantitative analysis of areas of particular importance to safeguard aircraft water integrity. In particular, the water service vehicle was identified as an implicating source of an increased microbial load within aircraft tanks.

This comprehensive study of aircraft water shows the diverse nature of bacteria that travel. However the bacteria represented do not fall into the dangerous infectious microorganism categories, e.g., Shiga toxin producing *E. coli*, *Legionella*, and *Enterococcus etc.*, which can inhabit water. Nevertheless, the isolated bacteria from this study have the potential to cause illness in certain sectors of the travelling population including immunocompromised individuals.
